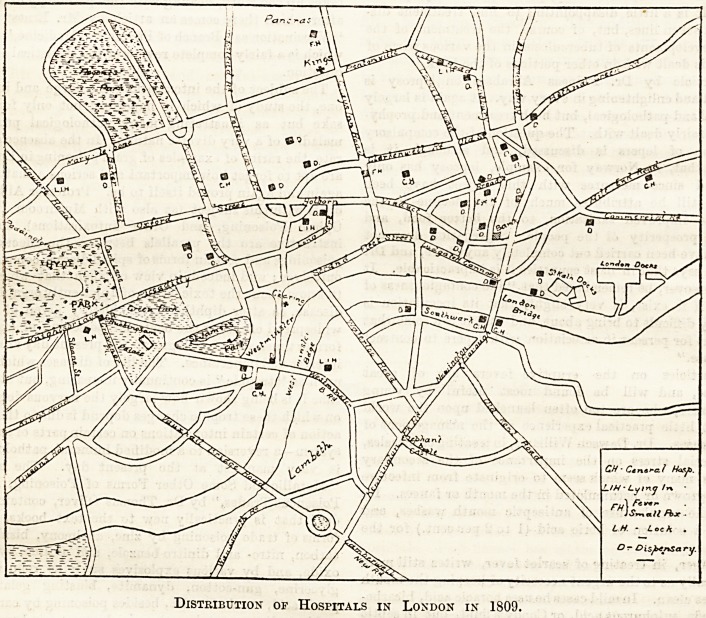# The Growth and Scope of Voluntary Hospitals in London

**Published:** 1897-09-25

**Authors:** 


### The Institutional Workshop.

### THE GROWTH AND SCOPE OF VOLUNTARY HOSPITALS IN LONDON.

(From a Correspondent.)

### X.—PROPORTION OF HOSPITALS TO POPULATION.

It is hard to conceive of London, as it was at the beginning of this century, unprovided with any of the machinery on which all friendly workers among the poor have grown to depend. Educational agencies touched but a small proportion of the children swarming in the streets, ready from want or idleness to drift into crime. Even so late as 1833 it was estimated that not half of the children in England received any education, and of those who did nominally attend school a large proportion went only on Sunday or in the evening. Often bound apprentices as mere babies to cruel masters, over-worked, untaught, savagely punished, ill-fed, the lot of the working man's child was hard indeed compared with the liberty and licence of the criminal classes. But we have not only to contemplate the removal of all the countless forces which in the present day are working for the well-being of the children; we have also to cut off all the assistance now wisely administered by the guardians of the poor, and nearly all the agencies which administer relief to special classes, if we would picture the condition of the poor in 1800. No lunatic asylums, no blind schools, no teaching for the deaf and dumb, no appliances for the cripple, no refuge for the incurable, no pensions for the aged except by private charity, no depôts for the supply of cheap food, no labour homes, no guilds, no benevolent funds or orphanages to each profession and trade, no clubs or baths or gymnasia or free libraries, no model dwellings, no savings banks or insurance societies for the working classes, little encouragement to keep a man on his feet, none to lift him up when he was down. Charity was universally administered in doles, in meat, coals, and soup tickets to the most clamorous. The workhouses were a disgrace to civilisation, and the enormous poor rates benefited only the administrators and a race of lazy beggars. It is not too much to say that at the opening of this century hospitals and dispensaries represented the only well-organised, heartily-supported agency for the relief of suffering in the metropolis. To what extent were they adequate for its needs?

Distribution of Hospitals in London in 1809.

The best data for determining the amount of relief afforded by the hospitals at this time is to be found in Highmore's "London Charities." The writer was a lawyer intimately acquainted with the management of more than one hospital, and possessed of the soundest views on hospital subjects. He devoted much pains to the elucidation of all questions relating to the relief of the poor in his day, and obtained information of a valuable character from almost every institution then existing. It will be convenient to take the year 1809, to which his investigations refer in the following computations, and the accompanying outline map may be taken as comprising the London of that day. Already the suburbs had begun to extend far out to the east and south; many wealthy residential quarters in the City had already been given up to warehouses or become thickly populated by the poor, and the practice of living "in the country"—at Camberwell, or Clapham—had become common even in the eighteenth century among City men of means, who were accustomed to pass a few days only of the week at their houses of business. It is not, therefore, possible to be very accurate in estimating the population of London. The census returns for 1800—the first regularly taken—stated it at about 959,000, within the limits of the map and for want of better data we must conclude that about one million was the number in 1809.

The number and position of all the institutions then in operation for the care of the sick poor is indicated on the map. They include 7 general hospitals, 4 lying-in hospitals, 2 for infectious diseases, 1 lock hospital, 1 eye hospital, 14 general dispensaries, 2 lying-in dispensaries, 5 vaccine lymph or cow-pock depôts, 3 truss or rupture dispensaries, 1 electrical dispensary, and 1 eye and ear dispensary. In all, then, there existed, for the benefit of the million Londoners in 1809, 7 general hospitals, 8 special hospitals, and 26 dispensaries of various sorts. In 1892, the population having increased to 4,221,452, the number of general hospitals was 22, the special hospitals amounted to 60, and the dispensaries to 57. Dividing the population among these institutions, the proportion works out as follows:—

| | Proportion of Population. 1809. | 1892. |
|---|---|---|
| To each general hospital ... ... | 142,857 ... | 196,429 |
| To each special hospital ... ... | 125,000 ... | 70,357 |
| To each dispensary ... ... ... | 38,461 ... | 74,062 |
| To each institution taken together ... | 24,390 ... | 30,370 |

London, then, has actually outgrown the accommodation available for its sick, and has never latterly reached the high-water mark of the first half of the century. In special hospitals alone, the spread of which is so marked a feature in hospital work during the present century, has the increase actually gained on the increase of population, and here it will be seen that nearly double the number of institutions is available in proportion to the population than in 1809.

Taking the commencement of the reign as a convenient epoch for studying the growth of population side by side with the extension of hospital work, we find the same tendency already at work. The population in 1837 amounted to 1,379,000; the institutions were 11 general hospitals, 12 special hospitals, 35 dispensaries. In all there were 58 institutions, so far as can be traced. The proportion of the population to each works out as follows:—

| | 1837. |
|---|---|
| To each general hospital ... ... | 125,363 |
| To each special hospital ... ... | 114,916 |
| To each dispensary ... ... ... | 39,400 |

So far then from having done too much in this direction it would seem at first sight that the London of to-day is actually in arrears in hospital construction. It will be necessary, however, to take into consideration not only the number of buildings, but the extent of the accommodation provided by each; not merely the population, but the average number of those who accept free medical relief, and in this direction some startling modifications of the above figures will be obtained.

## Figures and Tables

**Figure f1:**